# The “Dead-End Tract” and Its Role in Arrhythmogenesis

**DOI:** 10.3390/jcdd3020011

**Published:** 2016-04-05

**Authors:** Lennart de Vries, Astrid Hendriks, Tamas Szili-Torok

**Affiliations:** Department of Clinical Electrophysiology, Erasmus Medical Center, Rotterdam, 3015 CE, The Netherlands; l.j.devries@erasmusmc.nl (L.V.); a.a.hendriks@erasmusmc.nl (A.H.)

**Keywords:** cardiac conduction system, dead-end tract, idiopathic ventricular arrhythmia

## Abstract

Idiopathic outflow tract ventricular arrhythmias (VAs) represent a significant proportion of all VAs. The mechanism is thought to be catecholamine-mediated delayed after depolarizations and triggered activity, although other etiologies should be considered. In the adult cardiac conduction system it has been demonstrated that sometimes an embryonic branch, the so-called “dead-end tract”, persists beyond the bifurcation of the right and left bundle branch (LBB). Several findings suggest an involvement of this tract in idiopathic VAs (IVAs). The aim of this review is to summarize our current knowledge and the possible clinical significance of this tract.

## 1. Introduction

During the development of the ventricular conduction system sometimes a so-called “dead-end tract” is seen in addition to the right and left bundle branch, fading out on the crest of the muscular ventricular septum [1,2]. Remnants of the developing conduction system have been linked to the occurrence of arrhythmias [3]. The frequently described co-existence of VAs from the outflow tracts, the area in which the dead-end tract may persist, and the presence of atrioventricular re-entry tachycardias in structural normal hearts could implicate a clinical significance of this tract in the form of a connection between these regions [4,5,6,7,8]. Outflow tract VAs without underlying structural heart disease can be found in a large part of the population and can be very symptomatic [9,10,11,12,13,14,15,16]. The mechanism behind these IVAs is not completely understood and could be explained by the dead-end tract. In this review we aim to summarize our current knowledge and the clinical significance of this tract.

## 2. The Dead-End Tract in the Developing Cardiac Conduction System

### 2.1. The Developing Heart

The developing heart consists of cardiomyocytes with distinctive combinations of automaticity, conduction and contraction regulated by Tbox transcription factors [17,18]. Growth of the heart is established not by the division of myocytes, but by the addition of cells from a pool of precursors who do not obtain a definitive identity until they reach their final destination [19,20,21,22]. The formation of the cardiac chambers is characterized by ballooning: proliferation and differentiation in specific locations of the primary heart tube [23]. In the atrial appendages, trabeculated myocardium in the finalized heart is acquired from the ballooned atrial chambers [23]. The smooth part of the atrial walls is shaped from the myocardium from the connecting veins and the atrial component of the primary heart tube [24,25,26]. In the outer curve of the heart tube lies the origin of the developing ventricles [27]. After initial proliferation of trabeculated myocardium, compact myocardium is formed by discontinuation of proliferation at the luminal side and an increase in proliferation at the pericardial side [28,29,30].

### 2.2. The Developing Conduction System

#### 2.2.1. The Ring Theory

The ring theory, as proposed in earlier studies, states that four rings of specialized tissue precede the development of the conduction system [31]. Under normal circumstances these rings should, after fusing, lose their specialized quality or vanish by apoptosis. In the fully developed heart the sinus node, AV node, His bundle and bundle branches are thought to originate from the remnants of these rings [31]. This theory has been a source of great discussion. However, Lamers *et al.* provided a conclusive evaluation after studying material from human embryos showing that the inlet component of the morphologically right ventricle forms from the ascending limb of the embryonic ventricular loop, and that the inlet and apical trabecular elements of the muscular septum are formed from the same primary ventricular septum [32].

#### 2.2.2. Nodal Myocytes

When focusing specifically on the conduction system, the development starts in the early embryonic heart tube. Some early data on the development of the conduction of the heart came from studying avian embryos [33,34]. However, studies of human material were also available [32,35,36]. It is important to emphasize that during its development, the cardiac conduction system is not so much a single confined system as it is a composition of myocyte populations [37]. Although in the early embryonic stages ECGs resembling adult ones can be recorded, morphologically this arrangement of cells cannot be recognized [38,39].

Around Carnegie Stage 9–10 (19–23 days post fertilization), the first beats of the developing heart can be distinguished [40,41,42]. The first signs of the sinus node appear after five weeks of human development in the anteromedial wall of the right common cardinal vein [43,44]. This leading pacemaker at the most posterior part of the heart tube ensures a unidirectional peristaltic contraction wave. However, it is unclear how this pacemaker area remodels into a node distinct from the atrial neighboring myocardium. Due to the previously mentioned cardiomyocytes with their distinctive arrangement of characteristics, an adult-resembling ECG expressing the sequential activation of the atrial and ventricular chambers can now be acquired in the absence of electrical insulation or differentiated nodes and conduction system [45,46].

The slow conducting heart tube now evolves into separate atrial and ventricular myocardium segments characterized by higher conduction velocities [47,48,49,50]. At this time, in line with several observations, the heart tube itself is considered to be a conducting unit without a morphologically distinct conduction system complete with a pace-making sinuatrium, atrioventricular junctional tissue and an atrioventricular zone of slow conduction [51,52,53,54]. The transformation of this zone into a nodal structure starts to become visible from around five weeks of human development when the contrast of the primary nature of the nodal myocytes with the differentiating myocardium becomes more apparent over time [55,56]. The mechanism, similar to that of the transformation of the sinus node, remains unknown.

Responsible for the most distinctive trait of the early heart tube, varying slow- and fast-conducting areas, are the gap junctions enabling transfer of action potentials between myocytes [57]. The number and size of these gap junctions increase during development, however remain limited in the sinus- and atrioventricular node [43,58,59,60,61]. Composites of these membrane channels are the connexins of which five types are expressed in the human heart. Absent expression of these connexins, as is the case in nodal tissue, correlates with the absence of gap junctions and consequently with areas of slow conduction [62]. This characteristic has proven to be very helpful in distinguishing nodal from atrial cells [63,64].

#### 2.2.3. The Ventricular Conduction System

Current knowledge suggests that the ventricular conduction system may largely originate from the trabecular component of the ventricle [47,52,53,55,56,65,66,67,68,69,70,71,72,73,74,75,76,77,78,79,80]. As previously reported, the ventricular conduction system, as the rest of the conduction system, is theorized to originate from a primary ring of specialized cardiac tissue undergoing a series of changes in topography through the different stages of cardiac development [2,31]. Originating from myocardium tracing the primary interventricular foramen, part of this ring will eventually surround the subaortic outlet of the ventricle and the right atrioventricular junction just above the annulus [2]. The other part, responsible for conducting depolarizing impulses to the ventricles (the His bundle and bundle branches), hangs from the ventricular crest tracing the luminal side of the ventricles [2]. At this point it is unknown which regulatory pathways are responsible for the remodeling of these parts of the conduction system. It is thought that over time, insulation between the atrial and ventricular myocardium is accomplished by melding of the tissues of the atrioventricular sulcus with the atrioventricular cushions and that further separation of the ventricular conduction system from the surrounding myocardium may be regulated by cell-surface molecules which regulate cell-cell interactions [81,82].

### 2.3. Experimental Pathologic Evidence of the Dead-End Tract during Development

A great diversity in the position of conduction system cells exists within human hearts [83,84]. Of special interest are additional and remnant ventricular conduction branches that have previously been described [1,2,3,85].

After apoptosis or loss of “special” function of the earlier discussed rings, remnants may persist in the developed heart [3]. Sometimes consisting of entire branches, these remnants could be origins for re-entry, non-re-entry or automatic triggered arrhythmias [3].

In 1974, Anderson *et al.* described an additional right atrioventricular ring bundle in fetal human hearts [85]. Remnants of this ring were seen in infant and adult hearts, localized mostly anterolateral adjacent to the tricuspid orifice [85]. It was speculated that in some cases this remnant tissue might form a substrate for ventricular pre-excitation in the form of accessory atrioventricular pathways [85].

In a report by Kurosawa *et al.*, three cases were presented showing continuations of the conduction axis beyond the bundle branch bifurcation [1]. In this study two normal neonatal hearts and one with Fallot*’*s tetralogy were analyzed [1]. An extension starting on the summit of the ventricular septum after the bifurcation of the bundle branches was seen in these three sectioned hearts [1]. In the two normal hearts this extension reached the aortic root and close to the muscular summit of the septum where it faded out [1]. In the other heart it disappeared in the substance of the left ventricular aspect of the trabecular septum [1]. In this paper the tract was named a “dead-end tract” [1]. Their findings suggested that this dead-end tract was the more direct continuation of the conduction axis, as opposed to the right bundle branch [1]. The fact that they did not find this tract in adult hearts (in a previous study of 15 hearts, in subjects varying from stillbirth to adult age [86]), led them to suggest that it might only be seen in the neonatal and infant period [1]. This would imply that it represents a developmental stage in the maturation of the cardiac conduction axis [1].

Wessels *et al.* also demonstrated the dead-end tract as previously described by Kurosawa *et al.* [1,2]. Their findings showed an anterior continuation of cardiac specialized tissue of the atrioventricular bundle originating from the summit of interventricular septum, reaching into the retro-aortic root branch [2]. They also refer to a publication showing this continuation persists in guinea-pigs after birth [87].

Cells resembling atrioventricular junctional cells that were found along the AV orifices in two other reports, might provide additional clues for the presence of this tract [88,89].

## 3. Clinical Evidence of the Dead-End Tract

**Figure 1:**
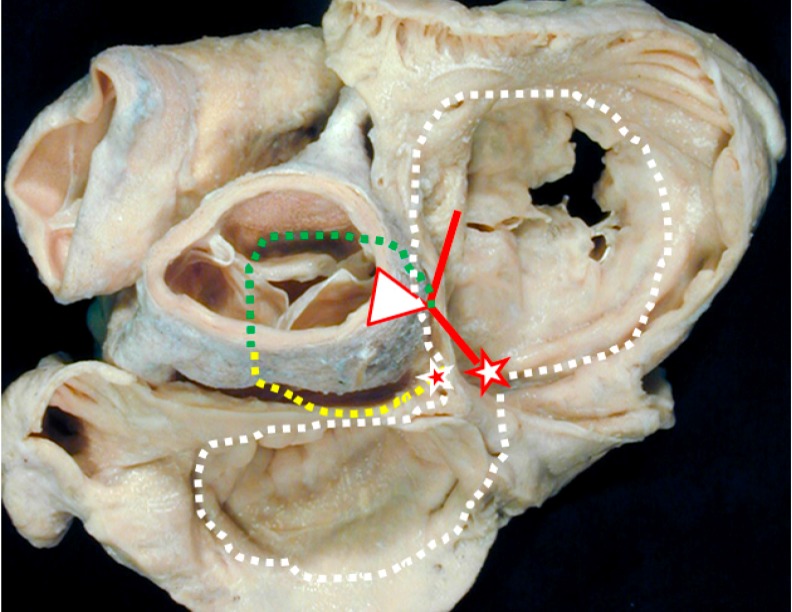
Cardiac base as seen from the atrial aspect. White star, red borders: the atrioventricular node; Red line: the bundle of His; Green dotted line: the dead-end tract (the continuation of the atrioventricular conduction axis); Yellow dotted line: the retro-aortic ring branch; White dotted line: embryonic atrioventricular ring; Red star, white borders: the retroaortic node; This image was both provided and labelled by Professor Robert H. Anderson and reproduced with his kind permission. Professor Anderson retains his intellectual copyright in the original image.

The arrhythmic potential of persistent embryonic tissue as discussed earlier may, in the case of the dead-end tract, become apparent in the form of idiopathic VAs. For example, the regularly seen co-existence of VA’s from the outflow tracts and the presence of accessory pathways (APs) or atrioventricular re-entry tachycardias in structural normal hearts has been observed in several previous reports [4,5,6,7,8]. These coinciding findings suggest there is a connection between these anatomically distant regions, which could be explained by the dead-end tract when taking into account the above mentioned pathological and topographical characteristics (Figure 1) [4,90,91,92].

**Figure 2:**
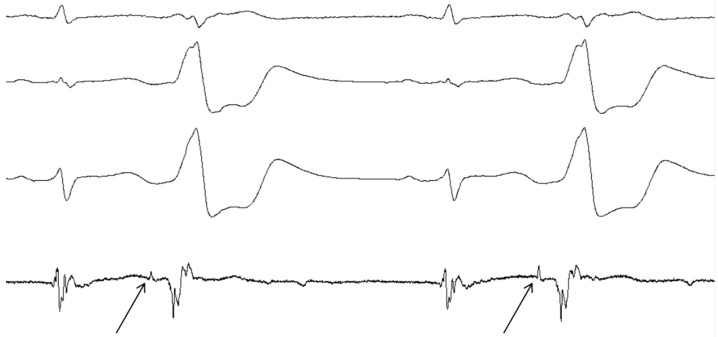
Pre-potentials on Intracardiac ECG. Intracardiac ECG of a patient from our center during catheter ablation of a VA originating from the AMC. Shown are a sinus complex followed by a PVC, which is then repeated. The arrows indicate the pre-potentials representing conduction over some kind of tract with an isoelectric interval of 92 ms.

Another important clue comes from several case reports and studies reporting so-called pre- or presystolic potentials as a target during catheter ablation [93,94,95,96,97,98,99,100,101,102,103,104,105,106,107]. These potentials with low amplitude, occurring slightly before the major potential were seen at target sites for ablation (Figure 2). In these studies ablation of idiopathic outflow tract, ventricular summit or aortomitral continuity (AMC) VT’s at the site of the pre-potentials was associated with a higher percentage of successful results. All of these structures lay within the route of the dead-end tract [1]. One study also reported a higher premature ventricular contraction (PVC) burden in patients with pre-potentials [93]. In most of these reports it was speculated that these pre-potentials could be caused by the presence of myocardial fibers, possibly representing the dead-end tract [93,97,98,100,102,104,105,106,107].

Exemplary for these studies is a report by Hachiya *et al.* in which successful ablation sites were located on the left or right coronary aortic sinus in 8.9% of outflow tract VAs [106]. In 9% of these cases a discrete pre-potential with a constant isoelectric interval was seen [106]. In all of these cases the site of successful ablation was at the pre-potential [106]. They described the pre-potential to be similar to that recorded between the His bundle and ventricular electrogram by electrodes in the His bundle area [106]. In support of the dead-end tract theory was the finding that the potential, seemingly originating from the normal conduction system, was recorded just beneath the successful ablation site [106]. This serves as a clue because it is known that ablation in the coronary aortic sinus does not affect the valve tissue itself, but instead ablates the myocardium of the ventricular septum roof just inferior to the valve [108,109]. Another observation they made was that after successful ablation they saw a delayed potential similar in morphology to the previously seen pre-potential, suggesting a shift in timing after ablation [106]. They hypothesized that the pre-potential represented the activation of a tract connecting the arrhythmia focus to the ventricular myocardium [106].

Additionally, some studies on ECG characteristics of IVAs revealed a delta wave-like onset of the QRS complex, which might serve as another hint at a possible source of these arrhythmias [96,110]. Delta waves often represent APs with slowed conduction and essentially, the dead-end tract might have similar properties to a slow conducting AP. One report, in addition to observed pre-potentials, described this delta wave-like onset in all of their 35 patients presenting with mitral annulus VA [96]. Another report demonstrated the delta wave-like onset in six of 48 IVA patients, most of them originating in the right ventricular outflow tract (RVOT), negatively associated with ablation success [110].

## 4. Discussion

### 4.1. Evidence

It is known that the occurrence of arrhythmias is related to certain preferential anatomical sites. As we have seen in the current report, persistence of embryonic remnants of the conduction system has been reported frequently [3,85,88,89]. More specifically, several pathological studies have demonstrated the dead-end tract in particular as a known anatomical entity [1,2,87]. These remnants could present a source of ectopic focal triggered activity and, provided that they are long enough to reach structures such as AMC or the outflow tracts, could contribute to re-entrant or non-reentrant circuits involving these regions.

### 4.2. Clinical Implications

IVAs can be found in 80% of the population [9,10], with 10% of all VT's accounting for idiopathic VT's, for the largest part originating from the outflow tracts [12]. Although generally considered benign, frequent ventricular arrhythmias can present a great burden on the patient and have been known to cause cardiomyopathy [12,13,14,15,16,111,112].

A higher quality of life and reversal of frequent ventricular arrhythmia associated cardiomyopathy has been accomplished after catheter ablation, making these arrhythmias an important target for treatment [13,113,114]. Since the mechanism behind these arrhythmias is not entirely clarified, other etiologies should be considered. When an anatomical substrate such as the dead-end tract can be targeted directly, this could theoretically improve ablation outcomes. As remarked earlier, targeting pre-potentials might provide higher success rates for these procedures.

### 4.3. Considerations and Limitations

Although many of the above-mentioned findings point to a possible role of and embryologic conduction tissue remnant as a cause for these arrhythmias, it is important to consider there is no direct evidence proving this involvement. The mechanism by which a remnant tract could cause outflow tract arrhythmias is not entirely clear. One should not rule out reentry as a possible mechanism. Furthermore, results suggesting involvement of the dead-end tract in the arrhythmia mechanism contain a significant amount of speculations. We do believe that for a better understanding and possible therapeutic improvements more basic research is needed. Even restudying the development of the human conduction system from the arrhythmogenesis point of view would be desirable. It seems that there is still a lack of consensus even on matters such as the timing of the appearance of the first morphological signs of the His bundle and bundle branches. Larger and more detailed pathological studies regarding the exact location and course of the dead-end tract should be performed to see whether it is able to reach the outflow tracts and AMC. Also, dense pace-mapping studies should be carried out to prove involvement of a common tract in the etiology of outflow tract ventricular arrhythmias and to assess its conduction properties. Finally, to clarify the actual prevalence of this possible entity, more population-based data needs to be collected.

## 5. Conclusions

The dead-end tract is a known embryological remnant of the developing ventricular conduction system. Pathological studies have shown us its existence and localization. In several publications a possible association between this tract and the occurrence of idiopathic ventricular arrhythmias, a very common and often impairing disorder in the general population, has been considered. Additional circumstantial evidence for the existence of this tract and its role in arrhythmogenesis can be found in the coincidence of disappearing outflow tract PVCs after AP ablation and the encounter of pre-potentials and a delta wave-like QRS onset at IVA ablation sites and the association of pre-potentials with higher success rates. More efficient targeting of this possible origin of IVAs could help to further improve ablation outcomes.
